# Influence of invasion history on rapid morphological divergence across island populations of an exotic bird

**DOI:** 10.1002/ece3.4021

**Published:** 2018-05-01

**Authors:** Rafael E. Valentin, Julie L. Lockwood, Blake A. Mathys, Dina M. Fonseca

**Affiliations:** ^1^ Department of Ecology, Evolution and Natural Resources Rutgers University New Brunswick NJ USA; ^2^ Division of Mathematics, Computer and Natural Sciences Ohio Dominican University Columbus OH USA; ^3^ Department of Entomology Rutgers University New Brunswick NJ USA

**Keywords:** Approximate Bayesian Computation, *Cardinalis cardinalis*, evolution, exotic species, founder effects, Hawaii, invasion history, morphology

## Abstract

There is increasing evidence that exotic populations may rapidly differentiate from those in their native range and that differences also arise among populations within the exotic range. Using morphological and DNA‐based analyses, we document the extent of trait divergence among native North American and exotic Hawaiian populations of northern cardinal (*Cardinalis cardinalis*). Furthermore, using a combination of historical records and DNA‐based analyses, we evaluate the role of founder effects in producing observed trait differences. We measured and compared key morphological traits across northern cardinal populations in the native and exotic ranges to assess whether trait divergence across the Hawaiian Islands, where this species was introduced between 1929 and 1931, reflected observed variation across native phylogeographic clades in its native North America. We used and added to prior phylogenetic analyses based on a mitochondrial locus to identify the most likely native source clade(s) for the Hawaiian cardinal populations. We then used Approximate Bayesian Computation (ABC) to evaluate the role of founder effects in producing the observed differences in body size and bill morphology across native and exotic populations. We found cardinal populations on the Hawaiian Islands had morphological traits that diverged substantially across islands and overlapped the trait space of all measured native North American clades. The phylogeographic analysis identified the eastern North American clade (*C. cardinalis cardinalis*) as the most likely and sole native source for all the Hawaiian cardinal populations. The ABC analyses supported written accounts of the cardinal's introduction that indicate the original 300 cardinals shipped to Hawaii were simultaneously and evenly released across Hawaii, Kauai, and Oahu. Populations on each island likely experienced bottlenecks followed by expansion, with cardinals from the island of Hawaii eventually colonizing Maui unaided. Overall, our results suggest that founder effects had limited impact on morphological trait divergence of exotic cardinal populations in the Hawaiian archipelago, which instead reflect postintroduction events.

## 1 INTRODUCTION

The recognition that biological invasions provide unique insight into the mechanics of evolutionary divergence has led to a spike in published research on postestablishment evolution of exotic species (e.g., Dlugosch & Parker, 2008a,b; Suarez & Tsutsui, 2008). There are now several examples of marked divergence in genetic or phenotypic traits between two or more exotic populations (Egizi, Fefferman, & Fonseca, 2015; Freed, Conant, & Fleischer, 1987; Lucek, Sivasundar, & Seehausen, 2014; Phillimore et al., 2008; Westley, Conway, & Fleming, 2012; Xu et al., 2010). Such differences can be explained by in situ adaptation to local biological and environmental conditions, or from events that occurred within the species’ invasion history (e.g., Allendorf & Lundquist, 2003; Dlugosch & Parker, 2008a; Keller & Taylor, 2008). In particular, founder effects can result in divergence of traits across exotic populations if colonizing individuals are derived from two or more genetically and/or phenotypically structured native subpopulations and introduced in such a way where these features are structured across the exotic range (Keller & Taylor, 2008). Here, we deduce the invasion history of northern cardinals (*Cardinalis cardinalis*) established across the main Hawaiian Islands and, using this history, evaluate the role of founder effects in producing previously observed morphological divergence of these populations (Mathys & Lockwood, 2011). In the process, we also elucidate the degree to which cardinals on Hawaii have diverged from their native source population(s), and provide insight into their postestablishment population dynamics.

**Figure 1:**
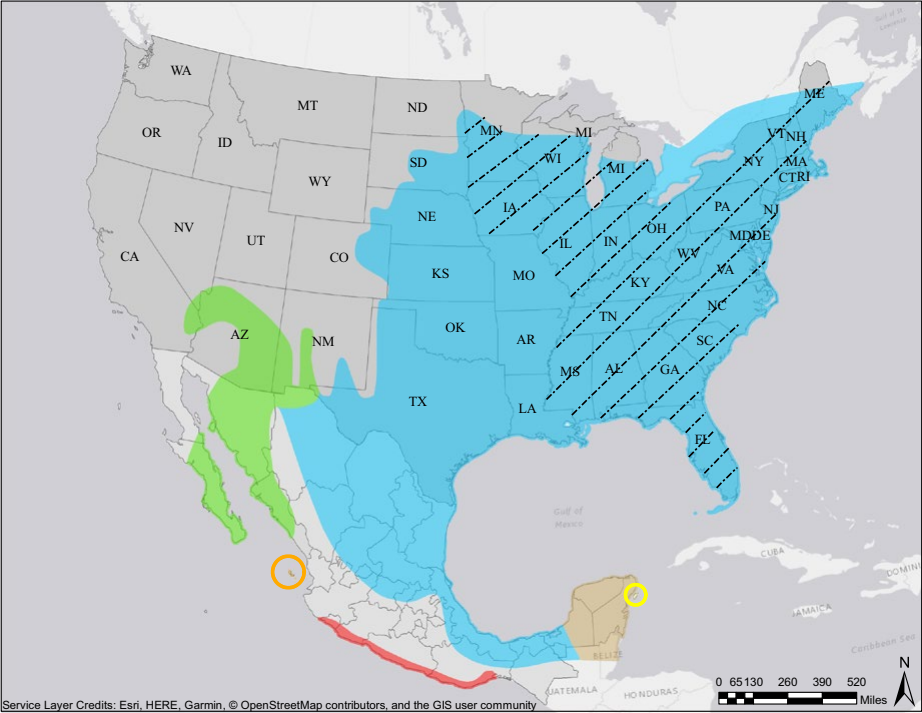
Map depicting the six northern cardinal (*Cardinalis cardinalis*) mitochondrial clades in their native range. Map adapted from Smith et al. (2011). Clades represented as follows: blue = *C.c. cardinalis*, green = *C.c. igneus*, orange = *C.c. mariae*, red = *carneus*, brown = *C.c. coccineus*, and yellow = *C.c. saturatus*. The textured portion of the blue clade represents the eastern region of the *C.c. cardinalis* clade, while the nontextured portion represents the western region

From written records, we know that between 1929 and 1931, 300–350 northern cardinals were purposefully transported and released onto Hawaii (Pyle & Pyle, 2009). These cardinals were shipped from the port of San Francisco (USA) and released onto Kauai, Oahu, and Hawaii Island (Pyle & Pyle, 2009). Northern cardinals are native to North America, with populations spanning the eastern half of the continent through to New Mexico and down into Mexico (Figure 1). There are six mitochondrial clades present in North America (Figure 1), with considerable morphological differences between them (Smith et al., 2011). The closest native population of northern cardinals to San Francisco is over 600 km to the south representing the *C.c. igneous* clade. There are no written records telling us whether the cardinals shipped from San Francisco came from this clade, or another one located further away but perhaps more connected to the city via train or other transportation mechanisms typical of this era. Thus, we do not know whether the cardinals throughout Hawaii were derived from one or more source clades; and, if more than one clade was involved, if a single or multiple clades founded the exotic populations on each island. The records also do not tell us how the 300–350 individual cardinals were divided across release events or how (or if) they were divided between shipments across years.

What we do know is this was the only introduction of northern cardinals to the archipelago, and they rapidly increased in population size after establishment, eventually colonizing all of the main Hawaiian Islands by the 1950s. We also know that current island cardinal populations are statistically different from each other in several morphological traits (e.g., wing and bill sizes—Mathys & Lockwood, 2011). These morphological traits are known to be heritable among birds (Badyaev & Martin, 2000a,b; Jensen et al., 2003), and Mathys and Lockwood (2011) show that observed across island differences are of such a magnitude that in situ genetic drift is not a likely causal mechanism (Mathys & Lockwood, 2011).

There are three ways invasion history could have produced the morphological divergence seen in cardinal populations across Hawaii. First, populations on each island may have been founded by individuals from genetically and morphologically distinct native source clades, and the morphological differences observed today recapitulate these across‐clade differences (Figure S1‐Scenario 1). Second, one or more island populations may represent an admixture of individuals sourced from different native cardinal clades (Figure S1‐Scenario 3). Any observed differences across islands today thus evolved in response to island‐specific selective forces enabled by the increases in genetic diversity that accompany admixture. Third, the cardinals on Hawaii may have been derived from one native source clade, which would suggest that current morphological differences arose after establishment from the existing genetic variation found within these founders (Figure S1‐Scenario 2).

We examine these possibilities by updating and expanding the between‐island morphological trait analysis from Mathys and Lockwood (2011). We then, for the first time, compare the distribution of traits across the Hawaiian Islands to traits typical of cardinal clades in the native range. Finally, we determine the most likely native source population(s) for the exotic island populations and deduce their postestablishment population dynamics using phylogeographic and Approximate Bayesian Computation (ABC) analyses. By combining these analyses, we assess which of the above three introduction and differentiation scenarios most likely occurred among northern cardinals in Hawaii, and we shed light on the postestablishment evolutionary dynamics of this species.

## 2 METHODS

### 2.1 Morphological analysis

In this analysis, we sought to establish the magnitude and direction of morphological differences in cardinals between the five main Hawaiian Islands, between the cardinals associated with each native range clade, and between the native clades and Hawaii. We used the following morphological traits: tail length, wing chord, culmen length, bill depth (at anterior margin of nares), and bill width (also at anterior margin of nares)—all measured in millimetres. These traits are commonly used metrics for evaluating evolutionary divergence between bird populations due to their known associations with life history and foraging adaptations (Lockwood, Moulton, & Anderson, 1993; Ricklefs & Travis, 1980).

We visited Kauai, Oahu, Maui, and Hawaii Island in the summer of 2008, and again in the summer of 2013, to obtain morphological measurements of 74 live‐caught northern cardinals. Mist nets were placed in areas that experience regular bird activity. No lures or baits were used in order to prevent bias in the sex ratio of captured individuals. Captured individuals were fitted with USGS numbered bands before release, allowing us to avoid measuring the traits of any one individual multiple times. All morphological measurements on field‐captured individuals were taken in the same season, thus avoiding systematic bias in morphological traits that vary with season (e.g., wing chord—Arendt & Faaborg, 1989). Only adults were measured, as young individuals are still growing and do not provide accurate measures of adult body dimensions. Culmen length, bill depth, bill width, and tarsus length were measured with a Mitutoyo dial caliper (Mitutoyo America Corporation, Aurora, IL, USA) to one‐hundredth of a millimetre precision. Tail length and wing chord were measured with a 15‐cm wing rule accurate to one millimetre (Avinet, Inc., Dryden, NY, USA).

In addition to live individuals, we measured specimens housed in the Bishop Museum (Hawaii, USA), American Museum of Natural History (New York, NY, USA), and the National Museum of Natural History (Washington, D.C., USA). In total, we measured 130 specimens collected across four of the six native range clades and 106 specimens collected on Kauai, Oahu, Maui, and Hawaii Island. All museum specimens of Hawaiian cardinals measured were collected between 1980 and 1999 and included both males and females. We did not have enough specimens measured from the *C.c. carneus* native clade, and none were available for the *C.c. mariae* clade, to include them in the analysis. We purposefully selected individual specimens that came from across the full geographic expanse of each of the four remaining native clades. Thus, for example in the case of the very widespread *C.c. cardinalis* clade (Figure 1), we measured individuals from Virginia, South Carolina, North Carolina, Montana, Missouri, Maryland, Washington, D.C., Ohio, Florida, Georgia, Texas, Michigan, New York, Kansas, and Mexico. This effort allowed us to capture a representative portion of the morphological trait variation within each native clade. All measurements were taken in the same way as for live specimens.

Data from live‐captured individuals and museum specimens were combined for all morphological analyses. We measured only museum specimens that were captured at the same time of year as the live‐caught individuals to reduce any systematic bias between the two data sources, and combined measurements for males and females to maximize sample sizes. Northern cardinals show very little differences between sexes in the traits we measured; however, to ensure that across‐population comparisons were not biased by sex‐specific differences, we kept sex ratios across clades and islands as close to 50:50 as possible.

Finally, it is well documented that bird specimens experience changes in some mensural characters (e.g., wing chord) after museum preparation (Bjordal, 1983; Haftorn, 1982; Winker, 1993) due to drying of the skin. In order to combine the measurements from live individuals with museum specimens, we multiplied field (live‐caught) measurements of tail length and wing chord by taxon and character‐specific correction factors following Winker (1993) and Mathys and Lockwood (2011). In order to correct for individuals with missing measurements due to condition of the specimen or inability to take all measurements in the field, we approximated missing trait values using the data imputation MICE package in R (Buuren & Groothuis‐Oudshoorn, 2010). This method was preferred as it has little impact on the observed population mean, uses the dataset itself to generate imputed data values, and does not reduce the variation in the dataset. We imputed trait information for <2% of the full dataset.

Recognizing that morphological traits are often intercorrelated, we collapsed the six measured traits from live‐caught and museum cardinals into two principal components using Principal Component Analysis (PCA; Lockwood et al., 1993) in R statistical software with the factoextra package (Kassambara & Mundt, 2016; Team, 2014). Prior to conducting the PCA, we log‐transformed all variables and then centered and scaled the means. The first two dimensions of the PCA (PC1 and PC2) explained 75% of the observed variation in morphological traits, with PC1 capturing overall size of individuals and PC2 reflecting the ratio of the bill to body size (Table S1). We retained the PC1 and PC2 scores for each measured individual so we could compare morphological differences across populations and clades.

We initially updated and expanded the between‐island morphological analysis of Mathys and Lockwood (2011) by increasing the number of individuals measured across islands and adding specimens from Maui to the comparisons. Using individual PC1 and PC2 scores, we evaluated differences in cardinal morphology between islands using multivariate analysis of variance (MANOVA) in R. If the overall MANOVA resulted in statistical significance, we followed that test with a series of pairwise MANOVA tests between islands.

Next, we compared the morphologies of Hawaiian cardinals to the four native cardinal clades for which we had sufficient data. To aid in visualizing quantitative differences in morphology across island populations and native clades, we plotted PC1 and PC2 for all measured cardinals in two‐dimensional space. We visually identified individuals from each native range clade using color‐coding, adding an ellipse that contained 95% of all individuals from these clades to clearly identify the range of morphologies present within each. We designated cardinals from Hawaii with a unique color code as well as designated individuals according to their island of residence using island‐specific symbols. This graph allows one to visualize the morphological “map” of native range cardinals, where each clade occupies a relatively distinct position in the two‐dimensional space, and then visually evaluate where the Hawaiian cardinals “fit” onto this map.

Using these data, we evaluated the following scenarios: (1) the Hawaiian cardinals fall entirely into the trait space of only one native range clade, indicating all Hawaiian cardinals were derived from this single native source and any divergence they show across islands is typical of the range of morphologies seen in that clade; (2) Hawaiian cardinals span two (or more) native range clade spaces and that cardinals from one island clearly fall within one clade and cardinals from another island fall in the other clade, indicating that interclade morphological differences are being recapitulated across islands (founder effect); or (3) the Hawaiian cardinals do not neatly fit into any single native clade's morphological space, indicating potential admixture at the time of introduction, postestablishment divergence, or both. We quantitatively evaluated differences in PC1 and PC2 between clades and Hawaii with MANOVA followed by pairwise MANOVA.

### 2.2 Sequence data generation

In order to determine the native source(s) of cardinal populations across Hawaii, we combined the *C. cardinalis* native range genetic data from Smith et al. (2011) and genetic information from the live‐caught individuals to create a merged northern cardinal dataset. Smith et al. (2011) used the sodium dehydrogenase subunit‐2 (ND2) mitochondrial locus to establish discrete genetic boundaries for six native range clades. In order to compare Hawaii cardinals with this dataset, we used the same locus. We collected feathers from 46 of the measured individuals caught in the Hawaiian Islands in 2013. Feathers were placed in small envelopes, and upon return to the lab, the calamus from multiple feathers was clipped to obtain a biological sample for each individual. These samples were placed in 1.5‐ml Eppendorf tubes in order to extract genomic material from cells found on the feather calamus. We extracted DNA using a DNeasy blood and tissue kit under standard protocols (Qiagen reference), with Proteinase K incubation taking place overnight (minimum of 8 hr) to ensure complete digestion.

We amplified 1,042 base pairs of ND2 via polymerase chain reaction (PCR) using primers L5215 (5′‐ TATCGGGCCCATACCCCGAAAAT‐3′) and HTrpC (5′‐ CGGACTTTACGACAAACTAAGAG‐3′), identical to those used by Smith et al. (2011). Amplification was accomplished with 20 μl reactions consisting of 1× PCR buffer (10 mmol/L Tris‐HCl, pH 8.3, and 50 mmol/L KCl), 2.25 mmol/L MgCL_2_, 150 μmol/L each dNTP, 200 nmol/L of each primer, 1 unit of Amplitaq Gold DNA Polymerase, and 3 μl of genomic DNA. The protocol was optimized to run at an initial denaturing temperature of 96°C for 10 min, followed by 40 cycles of the following steps: denaturing at 96°C for 45 s, annealing at 60°C for 30 s, and extension at 72°C for 45 s. Final extension was completed at 72°C for 5 min. All PCRs were run on a Veriti 96‐Well Thermal Cycler (Applied Biosystems, Life Technologies, Carlsbad, CA, USA). We visualized reactions in a 1% agarose gel with Ethidium Bromide and selected DNA fragments of appropriate size for sequencing. Successful amplicons were cleaned using ExoSAP‐IT (Affymetrix, OH), and mixes of 25pmoles of primer and 40 ng of template DNA were sent for cycle sequencing and sizing (Genscript, Piscataway, NJ, USA). Sequences were obtained using both primers to create a consensus of the full 1,042 bp ND2 sequence after chromatograms were cleaned and aligned in Sequencer 5.1 (GeneCodes, Ann Arbor, MI, USA). All sequences were evaluated for insertions and deletions, as well as translated to amino acids to check for stop codons and the presence of nuclear copies (Sorenson & Fleischer, 1996).

### 2.3 Phylogeographic analysis

We executed a phylogeographic analysis using the merged northern cardinal dataset to determine which native source clades were associated with each exotic island population. We ran the dataset through the program PartitionFinder 1.1.1 (Lanfear, Calcott, Ho, & Guindon, 2012) in Python v2.7 under two model schemes: unpartitioned whole gene ND2 sequences and partitioned by codon. We implemented a MrBayes model filter to select only the twenty‐four DNA evolutionary models that were compatible with the MrBayes program. PartitionFinder generated model schemes for both partitioned and unpartitioned data and ranked them by Akaike Information Criterion (AIC). We then constructed a phylogenetic tree with MrBayes v3.2.2 (Ronquist & Huelsenbeck, 2003) under the selected best scheme for both unpartitioned and partitioned data. The program was allowed to run for 10 million generations, while being sampled every 1,000, with a relative burn‐in of 0.25. We visually inspected MCMC chains using the program Tracer v1.6 (Rambaut, Suchard, Xie, & Drummond, 2014) to confirm adequate burn‐in and convergence of chains and used FigTree v1.4.2 (http://tree.bio.ed.ac.uk/software/figtree) for final tree assembly and inspection.

### 2.4 Approximate Bayesian computation analysis

We used Approximate Bayesian Computation (ABC) to test a suite of possible introduction and range expansion scenarios. Briefly, ABC is a Bayesian analysis that allows for direct comparison of multiple introduction hypotheses (known as scenarios) and provides relative probabilities for each, given the data provided. This comparison is accomplished by performing inference computations from simulated pseudo‐observed datasets (PODs) that take into consideration the putative introduction histories modeled, moving backward through time from the observed data. The PODs most similar to the observed dataset are then selected (with replacement) via a Euclidean distance measure (Cornuet et al., 2014; Estoup & Guillemaud, 2010; Lombaert et al., 2010; Valentin, Nielsen, Wiman, Lee, & Fonseca, 2017). The selected PODs have relative posterior probabilities calculated for their respective scenarios via a logistic regression estimate, allowing the user to select a significantly different scenario as being most likely to have occurred (Cornuet et al., 2014; Valentin et al., 2017).

We framed testable scenarios around three main questions: (1) Can we identify from which of the source clade(s) the cardinals brought to Hawaii (the founding cardinals) were sourced? (2) Can we assess if the 300+ cardinals that reached Hawaii were effectively divided into three evenly distributed groups of founders and released simultaneously across all three islands, or were approximately 100 founders introduced to a single island during each introduction event over 3 years? and (3) Can we identify which island population(s) provided the founders of the Maui population? For each question, we modeled two or more scenarios and then compared these against each other in order to quantify their relative probabilities. We used the program DIYABC to conduct these analyses (Cornuet et al., 2008, 2014) and used the following summary statistics to conduct our analyses: one‐sample statistics—number of haplotypes and number of segregating sites, two‐sample statistics—number of haplotypes.

To address the first question (source clade), we evaluated four variations of three scenarios. The first scenario supposed that the source of Hawaii cardinals was the western region of the source clade (see Results for clade analysis below; Figure 1). The second scenario supposed the source individuals were derived from the eastern region of the source clade (Figure 1). The third scenario supposed that the Hawaii population was a mix of both regions. For these three scenarios, the first variation evaluated which region was the likely source of the Hawaii introduction without enforcing a change in effective population size (i.e., no genetic bottleneck). The second variation reduced the effective population size after initial introduction into the Hawaiian archipelago (genetic bottleneck—conditioned to be less than both native sources) and then allowed the population to change (no condition set to Hawaiian populations). The last two variations (three and four) considered the possibility that each source region contained an unsampled population that was the source of Hawaii founders, and contains genetic haplotypes not present in our dataset. Variations three and four were identical to the above second and first variations, respectively, except an unsampled population for each region was used rather than the region data itself. The variation with the highest confidence in scenario choice (i.e., contained the least amount of error) and contained a statistically significant scenario was considered the most probable, given our data.

To address the second question (pattern of release events), we evaluated two variations of two scenarios. The first scenario supposed the 300+ cardinals transported from the mainland were equally divided among the three islands, but equal subsets were released in 1929, 1930, and 1931 resulting in smaller founding population sizes. The second scenario supposed that of the 300+ founding cardinals, roughly 100 were acquired and introduced to one island per year. We again evaluated whether there was evidence of a population bottleneck with our scenario variations. For the first variation, there was no change in effective population size enforced after founders were introduced to Hawaii (i.e., no enforced bottleneck—no restrictions placed on Hawaii parameters). For the second variation, we did enforce an initial reduction in effective population size (i.e., bottleneck—restricted Hawaii parameters to be less than native range and fit scenario) and then allowed the population to increase.

To address the third question (source of Maui cardinals), we evaluated three different scenarios: (1) colonizers to Maui came from Hawaii Island; (2) colonizers came from Oahu; and (3) colonizers were derived from both islands.

In all ABC scenarios, we set parameter priors to fit a uniform distribution (under default bounds) and placed conditions on parameter priors only to fit the intention of each scenario as defined above. We chose the HKY mutation model, based on the results from PartitionFinder during the phylogenetic analysis (see Results below, Table S2), and set it identically for all scenarios evaluated (Table 1). We ran all experiments for three million computations prior to conducting any analyses.

**Table 1:** Prior distributions used for all ABC analyses. Mutation parameters refer to selected DNA mutation model, distributions used, and bounds for said distributions within the model validation screen

Description	Prior distribution			
Mutation parameters				
Mutation model	HKY	10% invariant sites		Shape (2)
Mean mutation rate	Uniform	(min) 1.00E‐7	(max) 1.00E‐5	
Indiv. locus mutation rate	Gamma	(min) 1.00E‐7	(max) 1.00E‐5	Shape (2)
Mean coefficient (k C/T)	Uniform	(min) 1.5	(max) 20	
Indiv. locus coefficient (k C/T)	Gamma	(min) 1.5	(max) 20	Shape (2)

## 3 RESULTS

### 3.1 Morphological analyses

**Figure 2:**
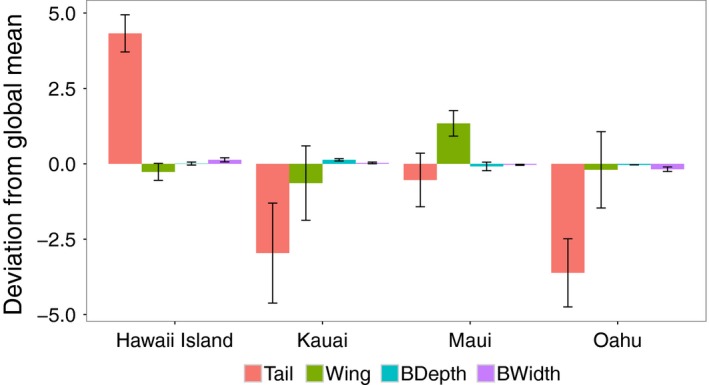
Morphological characteristics of northern cardinals sampled across five main Hawaiian Islands. The bars represent morphological traits read from left to right as: tail length (red), wing length (green), bill depth (blue), and bill width (purple). The zero‐line is the mean trait value calculated across all individuals and all islands. Deviations away from this value per island are depicted as bars, including calculated within‐island standard deviation for this trait. A large deviation from the island‐wide mean suggests that northern cardinal individuals sampled on that island have a divergent morphology. Most large differences across islands were due to tail and wing lengths

Reinforcing the findings of Mathys and Lockwood (2011), we found that northern cardinal populations showed substantial morphological divergence across the main Hawaiian Islands (Table 2, Figure 2). In particular, cardinals from Hawaii Island differ from those on all other islands except Maui (Table 2). Cardinals resident on Hawaii Island and Maui tend to be larger than their counterparts on Oahu and Kauai, especially in tail length (Figure 2). We also find residents of Maui have significantly larger wings than all other Hawaiian island populations (Figure 2).

**Table 2:** Results from the MANOVA analysis of northern cardinal morphological features taken across populations. Global results are the overall MANOVA testing for differences in PC1 and PC2 between the clades within the native range and Hawaii (grouped together), and the five main Hawaiian Islands. *p*‐values for MANOVA tests indicate overall significance across both PC1 and PC2, with individual PCs found significant highlighted in bold. Effect sizes for each PC are calculated using partial Eta^2^

Source	*n*	*df*	Approx. *F*	*P*	PC1 effect size	PC2 effect size
Hawaii (whole) & native range						
Global (PC1 & PC2)	229	4	31.91	<2.2e‐16	**0.32**	**0.40**
cardinalis × igneus	108	1	39.16	1.74E‐13	**0.41**	0.0094
cardinalis × coccineus	92	1	39.93	3.57E‐13	0.0040	**0.47**
cardinalis × saturatus	89	1	35.54	4.85E‐12	0.00014	**0.45**
igneus × coccineus	34	1	31.83	1.99E‐08	**0.44**	**0.55**
igneus × saturatus	31	1	26.04	2.78E‐07	**0.35**	**0.56**
coccineus × saturatus	15	1	0.28	.7634	0.011	0.029
Hawaii (whole) × cardinalis	189	1	40.89	1.81E‐15	**0.048**	**0.29**
Hawaii (whole) × igneus	131	1	53.36	<2.2e‐16	**0.36**	**0.14**
Hawaii (whole) × coccineus	115	1	11.28	3.39E‐05	**0.043**	**0.10**
Hawaii (whole) × saturatus	112	1	8.99	2.41E‐04	0.020	**0.10**
Hawaii only (by island)						
Global (PC1 & PC2)	103	3	2.95	.00882	**0.093**	0.041
Hawaii Island × Kauai	56	1	4.57	.01461	0.063	0.041
Hawaii Island × Maui	58	1	1.13	.329	0.038	0.003
Hawaii Island × Oahu	65	1	8.19	6.83E‐04	**0.14**	0.010
Kauai × Maui	38	1	1.75	.1878	0.0023	0.083
Kauai × Oahu	45	1	0.7388	.4835	0.021	0.022
Maui × Oahu	47	1	2.15	.1281	0.033	0.034

**Figure 3:**
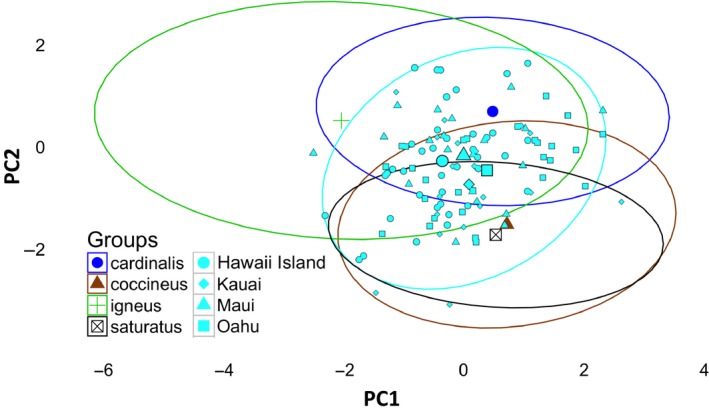
Two‐dimensional representation of northern cardinal morphological trait space using PC1 and PC2. PC1 reflects overall body size, whereas PC2 measures how bill depth and width change relative to a change in body size. We only include four of the native range clades in this analysis due to low sample size in two clades. Each oval encapsulates 95% of the variation in morphology between the individuals we measured, representing a clade‐specific trait space. Large symbols within each oval depict the mean PC scores for each clade. We depict all individual cardinals captured and measured in Hawaii in light blue (with different shapes for each island) to visually show the distribution of their morphology (light blue oval) relative to native clades

Our evaluation of morphological differences among the four evaluated phylogeographic clades confirms the existence of substantial morphological variation between northern cardinal clades across their native range (Table 2, Figure 3). In particular, we found that the native populations differ substantially in body size with *C.c. igneous* being the largest of the set, *C.c. cardinalis* moderately large sized, and the two lower‐latitude clades in Mexico the smallest (Figure 3). Bill dimensions also vary across clades, with cardinals exhibiting somewhat shorter and pointier bills (relative to body size) in the southern Mexican clades as compared to the two clades that cover sections of the United States (Figure 3). We found very little differentiation in morphology between the two southern clades *C.c. saturatus* and *C.c. coccineus*. This result agrees with ongoing research that indicates that the island clade of *C.c. saturatus* (located just off the Yucatan peninsula) is a recently derived population established via colonization of nearby *C.c. coccineus* individuals (Smith et al., 2011).

Collectively, the cardinals of Hawaii do not fall neatly into the morphological trait space of any single native clade (Figure 3). Hawaiian cardinals overlap in trait space with all four native clades for which we were able to obtain measurements. In addition, there is no clear pattern whereby the morphology of cardinals resident on an island corresponds to the morphology of cardinals from any one clade (Figure 3). Thus, there is no indication from this analysis that the pattern of morphological divergence observed on the islands matches any observed pattern of morphological differentiation among clades across the native range.

### 3.2 Sequence generation and phylogeographical analysis

After amplifying and sequencing the ND2 mtDNA locus for the 41 northern cardinal samples obtained from Hawaii, we found a total of 19 haplotypes (Table 3). We observed six, ten, and seven haplotypes for the cardinals present on Oahu, Hawaii Island, and Kauai, respectively, while Maui had just three haplotypes. These sequence data can be found in Genbank under accession numbers MH010209‐MH010303.

**Table 3:** Summary of number of samples (*n*.) used in the genetic analyses conducted, with localities sorted by mtDNA clade for the native range (with the west and east regions for *C.c. cardinalis* identified) and the Hawaiian archipelago. Each clade, and Hawaii, is further subdivided by locality, while providing the number of haplotypes per location (*n*. Haps) and the haplotypes observed. Any haplotypes followed by a number in parentheses indicates multiple specimens observed with said haplotype. Haplotypes in bold are those found only in Hawaii

Locality	*n*.	*n*. haps	Haplotypes
***C.c**. **cardinalis*** **(Total)**	**81**	**48**	
***C.c**. **cardinalis*** **(West)**	**54**	**32**	
Coahuila	7	6	4, 33(2), 34, 47, 48, 52
Kansas	5	4	24(2), 37, 48, 60
Louisiana	9	8	6, 19, 24, 26, 27(2), 30, 31, 46, 47
Oklahoma	10	7	22(2), 28, 41, 43, 45, 47 (2), 48 (2)
Tamaulipas/Nuevo Leon	8	8	5, 22, 32, 33, 38, 44, 50, 71
Texas/New Mexico	12	10	15, 22, 23, 27, 33, 47, 48(3), 53, 54, 61
Queretaro	2	2	36, 55
Veracruz	1	1	40
***C.c**. **cardinalis*** **(East)**	**27**	**20**	
New York	9	8	13, 18 (2), 20, 24, 31, 36, 47, 51
Florida/Georgia	9	7	10, 14, 17, 21, 22(2), 24, 49(2)
Minnesota/Wisconsin	9	7	11, 16, 25(3), 47, 48, 56, 62
***C.c**. **igneus***	**47**	**20**	
Arizona/New Mexico	11	2	73, 77(10)
Baja California	13	9	73, 74, 75, 76, 77, 83(5), 84, 88, 92
Sinaloa	19	13	72, 76, 77(6), 78, 80, 81, 82, 83, 85, 86(2), 87, 93, 95
Tiburón Island	4	3	79(2), 94, 95
***C.c**. **coccineus***	**11**	**3**	
Campeche	1	1	57
Yucatán	10	3	57(4), 58(5), 59
***C.c**. **carneus***	**8**	**3**	
Michoacán	6	1	2
Guerrero	1	1	1
Oaxaca	1	1	3
***C.c**. **saturatus***	**8**	**2**	
Cozumel Island			63(7), 64
***C.c**. **mariae***	**5**	**3**	
Tres Marías Islands			89(2), 90, 91(2)
**Hawaiian archipelago**	**41**	**19**	
Hawaii Island	14	10	**7**(2), 22, 27, **29**,** 39**(2), 47(2), 48, **66**,** 67**(2), **68**
Oahu	8	6	**12**(2), 24, **35**(2), **42**, 48, **69**
Kauai	8	7	**8**,** 9**(2), **12**, 24, **35**, 48, **70**
Maui	11	3	22(4), 24(4), **65**(3)

**Figure 4:**
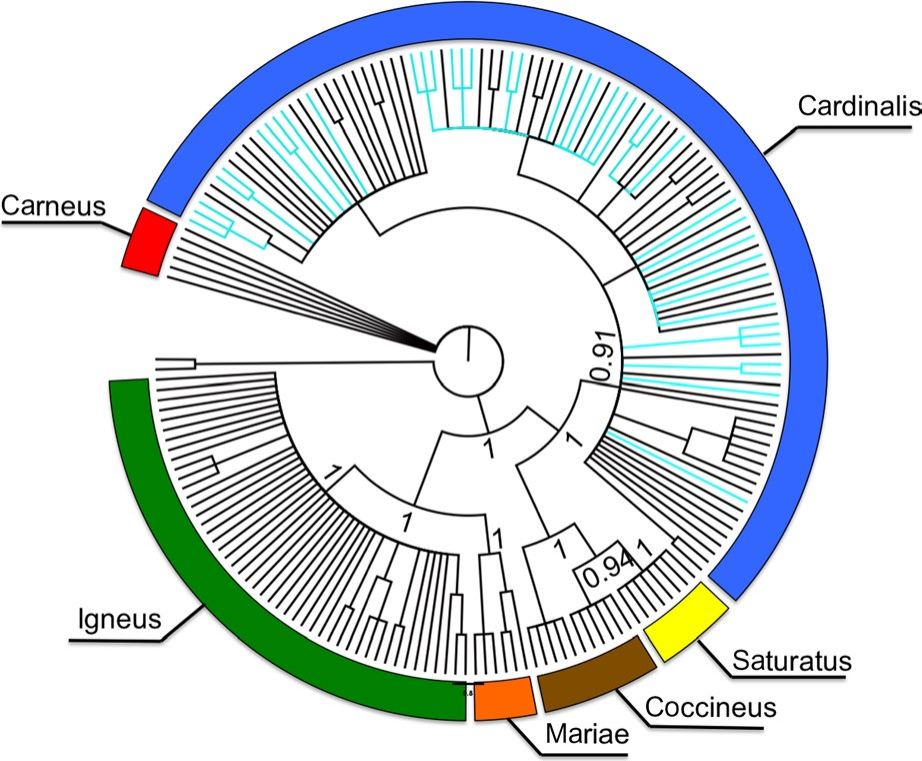
A phylogenetic breakdown of all native northern cardinal sequences analyzed by Smith et al. (2011), with the Hawaiian Islands samples we sequenced intermixed within the dataset. Monophyletic groups were categorized (and color‐coded) to their respective mitochondrial DNA clade, while the branches representing the Hawaiian individuals were color‐coded in light blue. All Hawaiian samples grouped with the *C.c. cardinalis* native range clade

After combining our sequence data with that of Smith et al. (2011), PartitionFinder 1.1.1 produced a single model scheme for unpartitioned data, with the GTR+I+G DNA evolutionary model. In contrast, data partitioned by codon position produced five model schemes, with the best scheme keeping the start codon for all three reading frames separated. For this scheme, the first and second codon positions were assigned the HKY + I evolutionary model, while the third codon position was assigned the GTR + G model (Table S2). After tree construction in MrBayes v3.2.2, and final assembly in FigTree v1.4.2, we found the unpartitioned scheme produced a tree showing a similar topology to that produced by Smith et al. (2011) (Figure 4). While of the 19 haplotypes found in the Hawaiian archipelago, 14 (74%) were not observed by Smith et al. (2011), all cardinal sequences from Hawaii fell into the *C.c. cardinalis* clade providing strong evidence that this was the single source clade for cardinals on Hawaii. The Hawaiian haplotypes were evenly distributed across the range of haplotypes in the *C.c. cardinalis* clade (Figure 4), which also did not show geographical assortment across the wide sampled range (Figure S2).

### 3.3 Approximate Bayesian computation analysis

The first question was aimed at identifying the region within the native source clade (*C.c. cardinalis*) from which the Hawaiian cardinals were derived. Without an enforced bottleneck, we did not find a significant difference in the relative probabilities among any of the three tested scenarios (Table 4). When we enforced a population bottleneck (variation two), however, we found the scenario where cardinals on Hawaii were derived from the eastern region of the *C.c. cardinalis* clade to be significantly more likely (Table 4). However, both of these variations had confidence scores below the remaining two, which included unsampled populations from each region. Of the remaining two variations, the third had the highest confidence score (0.643, Table 4), with the scenario where the source population came from an unsampled population within the eastern region of the *C.c. cardinalis* clade being the only one to be significant between the two variants (three and four) (0.8971 [0.6177, 1.000], Table 4).

**Table 4:** Probability and 95% credible interval for all Approximate Bayesian Computation scenarios used throughout the study, along with confidence in scenario choice. Variations in scenarios refer to no enforced reductions in the exotic population's effective population size (i.e., no bottleneck—variations 1 and 4), or enforced reductions (i.e., bottleneck) followed by a change in effective population that was free to either increase or decrease (variations 2 and 3)

Experiment	Prob.	95% CI	Conf.
*C.c cardinalis* source region (variation 1)			
1: Western source region	.3587	[0.3042, 0.4133]	0.511
2: Eastern source region	.2807	[0.2292, 0.3321]	
3: Western source + Eastern source	.3606	[0.3106, 0.4105]	
*C.c cardinalis* source region (variation 2)			
1: Western source region	.2576	[0.2203, 0.2949]	0.501
2: Eastern source region	.4451	[0.4048, 0.4854]	
3: Western source + Eastern source	.2973	[0.2509, 0.3437]	
*C.c cardinalis* source region (variation 3)			
1: Western unsampled source region	.0638	[0.0000, 0.2523]	0.643
2: Eastern unsampled source region	.8971	[0.6177, 1.0000]	
3: Western + Eastern unsampled source	.0391	[0.0000, 0.1722]	
*C.c cardinalis* source region (variation 4)			
1: Western unsampled source region	.4016	[0.0000, 1.0000]	0.596
2: Eastern unsampled source region	.0000	[0.0000, 1.0000]	
3: Western + Eastern unsampled source	.5984	[0.0000, 1.0000]	
Hawaii introduction scheme			
1: Introduced to Hawaii evenly (no bottleneck enforced)	.4088	[0.3322, 0.4853]	0.604
2: Introduced to Hawaii structured (no bottleneck enforced)	.0018	[0.0000, 0.0780]	
3: Introduced to Hawaii evenly (bottleneck enforced)	.5879	[0.5344, 0.6415]	
4: Introduced to Hawaii structured (bottleneck enforced)	.0015	[0.0000, 0.0777]	
Maui introduction scheme			
1: Colonized from Hawaii Island	.5369	[0.5059, 0.5679]	0.491
2: Colonized from Oahu	.1501	[0.1309, 0.1692]	
3: Colonized from both	.3130	[0.2845, 0.3415]	

Regarding the second question, results also indicated little genetic support for the transported founders having been released on each island across several years, regardless of the presence of bottlenecks (0.0015 [0.000, 0.0777]—effective population held static, and 0.0018 [0.000, 0.0780]—effective population bottleneck followed by increase). The scenario where cardinals were released simultaneously and evenly across islands proved most likely, and the scenario that included bottlenecks (prob = 0.5879 [0.5344, 0.6415]) was significantly more likely than the scenario where a bottleneck was not enforced (prob = 0.4088 [0.3322, 0.4853]).

Regarding the third question, we found the scenario where individuals from Hawaii Island colonized Maui had a higher relative probability (prob = 0.5086 [0.4636, 0.5537]) than the scenario were Maui colonizers were derived from Oahu (prob = 0.1508 [0.1226, 0.1789]), or from both Oahu and Hawaii Island (prob = 0.3406 [0.2982, 0.3831]).

## 4 DISCUSSION

A species’ invasion history can profoundly influence the degree of divergence observed, including via founder effects whereby phenotypic and genetic spatial structure in the species’ native range is captured and then recapitulated across exotic populations (Keller & Taylor, 2008). Here, we combined a suite of morphological and genetic analyses to deduce the invasion history of northern cardinals on Hawaii, including directly testing for the presence of founder effects in producing between‐island phenotypic variation. In total, we found evidence of an intricate history of colonization, spread, and postinvasion morphological differentiation.

Our phylogenetic tree indicated that the *C.c. cardinalis* native clade was the only source of individuals introduced to the Hawaiian Islands. Furthermore, the ABC analyses gave the highest likelihood to the scenario where founding individuals were derived from populations in the eastern half of that clade. San Francisco was an active commercial center in the 1920s and 1930s so we suspect that the 300 to 350 founder cardinals were captured near a large city in the eastern portion of the United States and shipped by train to San Francisco.

Furthermore, we found that only about half the measured individuals from Hawaii fell within the morphological variation we documented across the *C.c. cardinalis* clade. As all cardinals now resident on Hawaii were likely derived from individuals sourced from that native clade, the morphological analyses make clear that the observed morphological divergence of cardinals on Hawaii is not the result of founder effects. The cardinals in Hawaii that exceeded the *C.c. cardinalis* trait space fell mostly within the southeastern clades of *C.c. coccineus* (a clade within the southeastern peninsula of Mexico) and *C.c. saturatus* (an island population derived from *C.c. coccineus*). There is no evidence that northern cardinal populations in eastern North America have evolved over the time span that cardinals have been resident in Hawaii. Thus, although circumstantial, this evidence suggests that cardinals on Hawaii have diverged in morphology away from their native continental source population toward body sizes and bill shapes that are more typical of island and peninsular cardinal populations.

Furthermore, we found that cardinal populations on Kauai, Oahu, and Hawaii Island were likely all simultaneously founded by equal numbers of transported individuals and that all of these founder populations experienced a bottleneck. While many of the haplotypes present among Hawaii cardinals were not present in the *C.c. cardinalis* clade sequences, the ND2 locus sampled exhibited very high levels of diversity (48 haplotypes in 78 specimens, Smith et al., 2011). Therefore, the ABC analysis suggested that these haplotypes likely originated from unsampled haplotypes within the eastern region of the source clade. While it is possible some of the haplotype variants could have emerged postintroduction through mutations (e.g., Agrawal & Wang, 2008; Kaňuch, Berggren, & Cassel‐Lundhagen, 2014; Vandepitte et al., 2014), such mutations would likely have added only a few new haplotypes and not likely the 74% new haplotypes we detected. With a substantial increase in sampling across the *C.c. cardinalis* clade, we suspect a number of these haplotypes would be found, and from this information, it may be possible to further resolve the source population of Hawaiian cardinals.

Finally, the ABC analyses demonstrated a strong likelihood that the Hawaii Island cardinal population was the source of cardinals now resident on Maui. This scenario is supported by the fact that cardinals on Maui do not differ in overall morphology from those found on Hawaii Island. However, we do find that Maui cardinals have larger wings than cardinals on the other islands. The larger wing size in Maui cardinals could have resulted from selection on founders, as there is no record that humans mediated the expansion of cardinals to Maui. If so, this might be the only evidence of founder effects in Hawaiian cardinals.

Based on this collection of evidence, the story of the establishment and divergence of northern cardinals on Hawaii seems to be as follows. In the early 1930s, 300 to 350 cardinals were captured in the eastern United States and shipped to San Francisco likely by train. These individuals were then shipped by boat to the Hawaiian archipelago and released simultaneously and in approximately equal numbers on Hawaii Island, Kauai and Oahu. A subset of these individuals founded viable exotic populations on all three islands. At a later date, individuals from Hawaii Island colonized Maui. As these initial founding events cardinals have substantially diverged in morphology from their native source clade, and within the islands, cardinals on Hawaii Island and Maui show particularly divergent morphologies compared to the other islands.

Our results add to a growing number of studies that demonstrate evolution within an invasive species’ new range (Egizi et al., 2015; Whitney & Gabler, 2008). Most questions now center on deducing the mechanisms driving these patterns, using these examples to inform our broader understanding of evolutionary diversification processes. Relative to the evolution of morphological traits among birds colonizing islands, likely mechanisms of divergence center on factors such as thermoregulation, competition, and predation all of which can vary substantially on islands as compared to a mainland (Duncan & Blackburn, 2002; Luther & Greenberg, 2014; Moulton & Lockwood, 1992; Moulton, Sanderson, & Labisky, 2001). Our approach combining detailed historical records, comprehensive phenotypic analysis, and rigorous phylogenetic and population genetic techniques allowed us to reveal insights into the mechanisms that have produced postinvasion divergence in this exotic bird. Aside from conducting a full genomic or transcriptomic analysis of northern cardinals on Hawaii, however, we cannot at this point determine which of these potential mechanisms has driven the evolution of exotic cardinals in Hawaii.

## CONFLICT OF INTEREST

None declared.

## AUTHOR CONTRIBUTION

REV performed all laboratory work, collected morphological data, performed all morphological and genetic analyses, and wrote the manuscript. JLL collected morphological data, oversaw all morphological analyses, and wrote the manuscript. BAM collected morphological data, collected biological material for laboratory work, and wrote the manuscript. DMF oversaw all laboratory work, oversaw all genetic analyses, and wrote the manuscript.
