# Supplementary material for: Influence of invasion history on rapid morphological divergence across island populations of an exotic bird
Source: Ecol Evol. 2018 May 1;8(11):5291–302. doi: 10.1002/ece3.4021 (PMC6010901; doi:10.1002/ece3.4021)
Supplement: Supplementary file 1 [file ECE3-8-5291-s001.docx]

**Influence of invasion history on rapid morphological divergence across island populations of an exotic bird**

Influence of invasion on diverged populations

Rafael E. Valentin^1,4^, Julie L. Lockwood^1^, Blake A. Mathys^2^ and Dina Fonseca^1,3^

^1^Department of Ecology, Evolution and Natural Resources, Rutgers University, New Brunswick, NJ USA

^2^Division of Mathematics, Computer and Natural Sciences, Ohio Dominican University, Columbus, OH, USA

^3^Department of Entomology, Rutgers University, New Brunswick, NJ USA

^4^**Corresponding author**: Rafael E. Valentin, Ecology and Evolution Graduate Program, 14 College Farm Rd., New Brunswick, NJ 08901, USA; Rafael.Valentin@Rutgers.edu

Keywords: Legacy effects, *Cardinalis cardinalis*, Hawaii, morphology, Approximate Bayesian Computation, exotic species, diversification, genetic

Original Research

**Figure S1**. Three scenarios describing the introduction of northern cardinals to the main Hawaiian Islands. Scenario I (two gray lines) = individual cardinals were collected from two clades, and the individuals of one clade were introduced to a subset of all islands whereas the individuals of the other clade were introduced to a separate set. Scenario II (black line) = all individuals collected from the native range come from a single clade and they were released across all islands. Scenario III (blue line) = individuals were collected from more than one native clade but all were introduced to every island, effectively creating an admixed population on each island. We have depicted specific clades as sources, and islands as sets where introduction occurred, for illustration only. We have no reason to *a priori* restrict the set of clades, or the introduction scenarios, considered within our analyses.


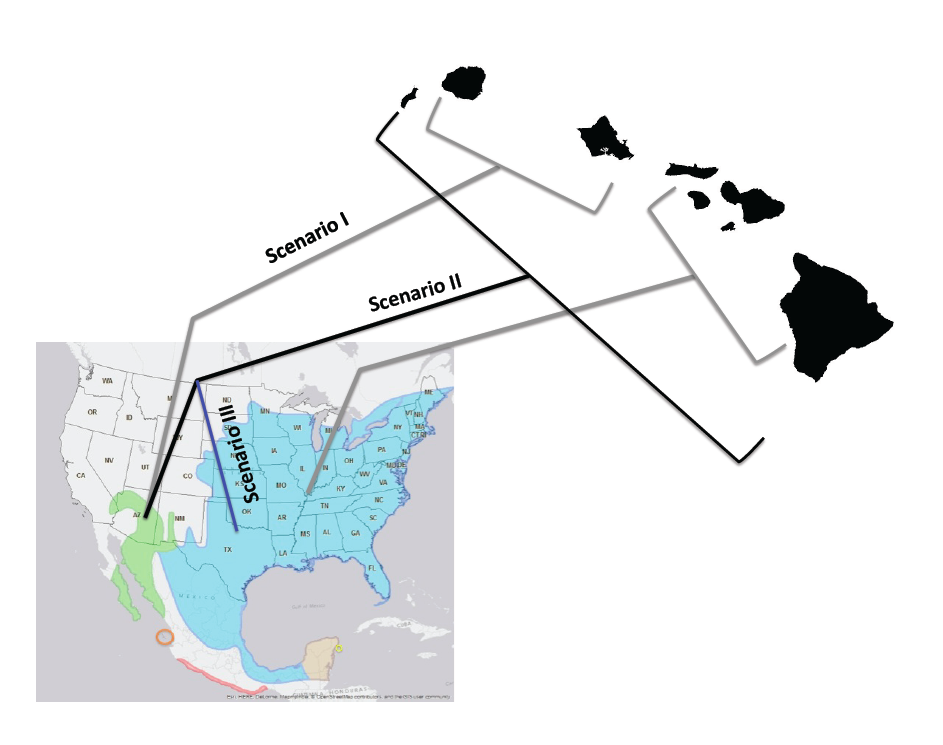


**Figure S2**. Haplotype network for *C.c.cardinalis* and Hawaiian cardinals. Network nodes in black indicate haplotypes found in *C.c.cardinalis*, while nodes in blue are those found in Hawaii. Numbers in bold indicate haplotypes seen both in Hawaii and across the *C.c.cardinalis* native range, while black nodes with a blue numbers (i.e. 22, 24, 47) indicate haplotypes that are seen in either eastern and western regions of the *C.c.cardinalis* clade and in Hawaii. Numbers in red indicate haplotypes found in the western region of the *C.c.cardinalis* range, while numbers in black (not next to blue nodes) indicate haplotypes found in the eastern region of *C.c.cardinalis* range. Numbers in purple indicate haplotypes seen in both the eastern and western regions.


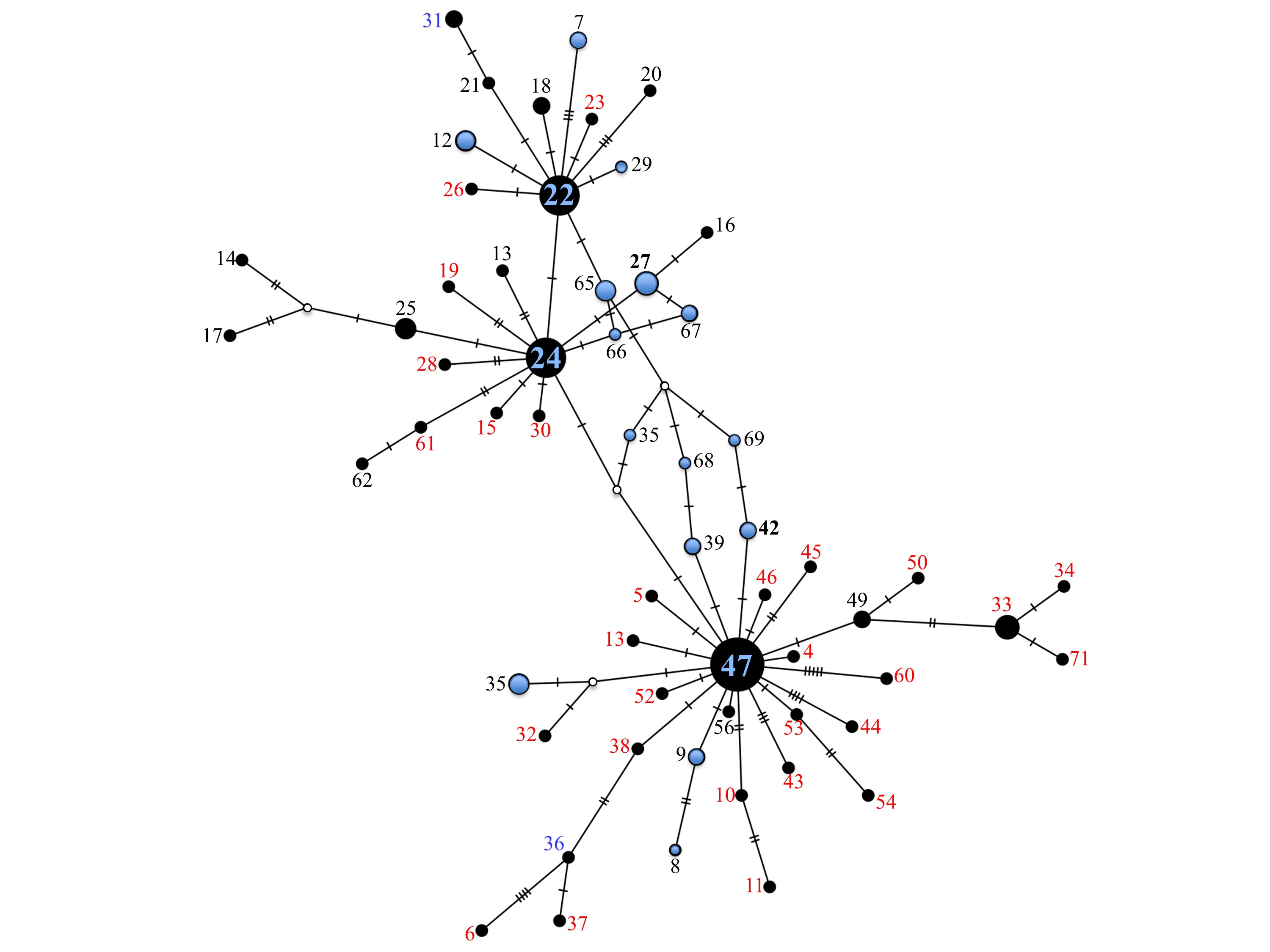


**Table S1**. Principle Component Analysis (PCA) outputs for the combined native range and Hawaiian northern cardinal (all islands in one group) morphological dataset. The top table provides eigenvalues and the amount of morphological variation explained by each principle component (PC). The bottom table provides the PC loadings (correlation) for each morphological trait measured.

| PCA Summary | | |
| --- | --- | --- |
|  | **Eigenvalue** | **% Variance** |
| PC.1 | 1.79692 | 44.923 |
| PC.2 | 1.19142 | 29.7855 |
| PC.3 | 0.57145 | 14.2864 |
| PC.4 | 0.44021 | 11.0052 |

| PC Loadings | | | | |
| --- | --- | --- | --- | --- |
|  | **PC.1** | **PC.2** | **PC.3** | **PC.4** |
| Tail Length | -0.49524 | 0.46083 | 0.71721 | -0.16728 |
| Wing Length | -0.4038 | 0.60789 | -0.68166 | -0.05252 |
| Bill Depth | -0.58893 | -0.34461 | -0.01477 | 0.73089 |
| Bill Width | -0.49482 | -0.54713 | -0.14398 | -0.65959 |

**Table S2**. MrBayes specific mutation models for the phylogeographic analysis found to be best via the program PartitionFinder given the specific codon partitioning schemes. Models were partitioned by codon scheme, and the best model(s) for each were selected and scored via AIC.
